# Towards the uniform distribution of null *P *values on Affymetrix microarrays

**DOI:** 10.1186/gb-2007-8-5-r69

**Published:** 2007-05-01

**Authors:** Anthony A Fodor, Timothy L Tickle, Christine Richardson

**Affiliations:** 1 Bioinformatics Resource Center, The University of North Carolina at Charlotte, University City Boulevard, Charlotte, North Carolina 28223, USA; 2 Department of Biology, The University of North Carolina at Charlotte, University City Boulevard, Charlotte, North Carolina 28223, USA

## Abstract

Methods to control false-positive rates require that *P *values of genes that are not differentially expressed follow a uniform distribution. Commonly used microarray statistics can generate *P *values that do not meet this assumption. We show that poorly characterized variance, imperfect normalization, and cross-hybridization are among the many causes of this non-uniform distribution. We demonstrate a simple technique that produces *P *values that are close to uniform for nondifferentially expressed genes in control datasets.

## Abstract 2

Estimating the *P *value from the overall distribution of scores on the microarray can produce *P *values that are much closer to a uniform distribution.

## Background

Microarray data typically involve tens of thousands of genes but only a handful of replicates. It is therefore difficult to establish appropriate *P *value thresholds for significance. For example, consider the response of 40,000 genes to two different experimental conditions, say diseased and healthy tissue. If a significance level of *P *< 0.05 is chosen, then one would expect an unacceptable number (2,000 [40,000 × 0.05]) of false positives. A conceptually simple procedure, the Bonferroni correction, would set a threshold of *P *= 1.25 × 10^-6 ^(0.05/40,000). Using this *P *value as the threshold for significance, there is only a 0.05 chance of any false positives across all of the 40,000 comparisons between the two conditions. Such metrics are said to control the 'family-wise error rate'. Family-wise error rate is often assumed to be too conservative for microarray experiments, because there are often no results with *P *values below the threshold for the modest number of samples that make up most microarray experiments. Recently, 'false discovery rate' (FDR) was proposed as an alternative, more permissive approach to estimating significance of microarray experiments [1-4]. This metric acknowledges that biologists are often able to tolerate some error in gene lists. For example, a FDR could be set at 10%, in which case a list of 100 genes would be expected to have as many as 10 false positives.

No matter what threshold is used to control significance in microarray experiments, there is an inherent assumption that the *P *values of genes that are not differentially expressed follow a uniform distribution. For example, genes that are not differentially expressed should have a *P *value of 0.01 or smaller only 1% of the time. The uniform distribution of null *P *values seems like a safe assumption that is guaranteed by the laws of statistics. However, if for some reason this assumption is not met, then attempts to determine a threshold of significance may yield meaningless results [2,5].

In this report we show that commonly used statistics can in fact generate distributions of *P *values for non-differentially expressed genes that are far from uniform. We demonstrate a simple method for producing *P *values that are much closer to the expected uniform distribution.

## Results and discussion

### RMA summation and quantile-quantile normalization suppress the pooled variance of each gene

Our central argument is that it is a rational choice to assume that, when comparing two conditions, the pooled variance of each gene on the array is approximately constant. If this assumption is true, then the distribution of scores under a *t *test or variant approaches the normal distribution. We begin our assertion that this assumption is reasonable by examining a control dataset released by Affymetrix. The Affymetrix HG-U133A Latin Square dataset consists of 14 'experiments' (labeled 1 to 14), each with three replicates. Each of the 14 experiments contains 42 genes that are spiked in at known concentrations against a constant background of human RNA. Of the approximately 22,000 genes on the chip, the only ones that should be different when comparing across experiments are the 42 genes that were spiked in at different concentrations. We shall refer to genes that were not spiked in as null genes, because the null hypothesis of equal expression in all conditions is true for these genes.

For two experimental conditions with sample sizes in each condition n_1 _and n_2_, we have our usual definition of a *t *test assuming equal variance:

t=x¯1−x¯2σ2
 MathType@MTEF@5@5@+=feaafiart1ev1aaatCvAUfeBSjuyZL2yd9gzLbvyNv2Caerbhv2BYDwAHbqedmvETj2BSbqee0evGueE0jxyaibaiKI8=vI8tuQ8FMI8Gi=hEeeu0xXdbba9frFj0=OqFfea0dXdd9vqai=hGuQ8kuc9pgc9s8qqaq=dirpe0xb9q8qiLsFr0=vr0=vr0dc8meaabaqaciGacaGaaeqabaqadeqadaaakeaaieaacaWF0bqefeKCPfgBaGabaiaa+bcaiiaacqqF9aqpcaGFGaWaaSaaaeaaceWF4bGbaebadaWgaaWcbaGaa8xmaaqabaGccqqFsislceWF4bGbaebadaWgaaWcbaGaa8NmaaqabaaakeaadaGcaaqaaGGaciab8n8aZnaaCaaaleqabaGaa8Nmaaaaaeqaaaaaaaa@401B@

σ2=∑j=1n1(xj−x¯1)2+∑j=1n2(xj−x¯2)2n1+n2−2(1n1+1n2)
 MathType@MTEF@5@5@+=feaafiart1ev1aaatCvAUfeBSjuyZL2yd9gzLbvyNv2Caerbhv2BYDwAHbqedmvETj2BSbqee0evGueE0jxyaibaiKI8=vI8tuQ8FMI8Gi=hEeeu0xXdbba9frFj0=OqFfea0dXdd9vqai=hGuQ8kuc9pgc9s8qqaq=dirpe0xb9q8qiLsFr0=vr0=vr0dc8meaabaqaciGacaGaaeqabaqadeqadaaakeaaiiGacqWFdpWCdaahaaWcbeqaaGqaaiaa+jdaaaGccqGH9aqpdaWcaaqaamaaqahabaaccaGae0hkaGIaa4hEamaaBaaaleaacaGFQbaabeaakiabgkHiTiqa+HhagaqeamaaBaaaleaacaGFXaaabeaakiab9LcaPmaaCaaaleqabaGaa4NmaaaaaeaacaGFQbGaeyypa0Jaa4xmaaqaaiaa+5gadaWgaaadbaGaa4xmaaqabaaaniabggHiLdGccqGHRaWkdaaeWbqaaiab9HcaOiaa+HhadaWgaaWcbaGaa4NAaaqabaGccqGHsislceGF4bGbaebadaWgaaWcbaGaa4NmaaqabaGccqqFPaqkdaahaaWcbeqaaiaa+jdaaaaabaGaa4NAaiabg2da9iaa+fdaaeaacaGFUbWaaSbaaWqaaiaa+jdaaeqaaaqdcqGHris5aaGcbaGaa4NBamaaBaaaleaacaGFXaaabeaaruqqYLwySbaceaGccaaFGaGae03kaSIaaWhiaiaa+5gadaWgaaWcbaGaa4NmaaqabaGccaaFGaGaeyOeI0Iaa4NmaaaadaqadaqaamaalaaabaGaa4xmaaqaaiaa+5gadaWgaaWcbaGaa4xmaaqabaaaaOGaey4kaSYaaSaaaeaacaGFXaaabaGaa4NBamaaBaaaleaacaGFYaaabeaaaaaakiaawIcacaGLPaaaaaa@65EF@

**Figure 1:**
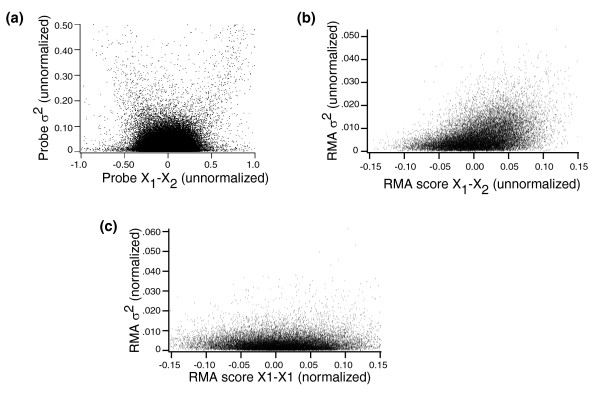
Standard error as a function of the difference in means. Shown is *σ*^2 ^as a function of x¯1−x¯2
 MathType@MTEF@5@5@+=feaafiart1ev1aaatCvAUfeBSjuyZL2yd9gzLbvyNv2Caerbhv2BYDwAHbqedmvETj2BSbqee0evGueE0jxyaibaiKI8=vI8tuQ8FMI8Gi=hEeeu0xXdbba9frFj0=OqFfea0dXdd9vqai=hGuQ8kuc9pgc9s8qqaq=dirpe0xb9q8qiLsFr0=vr0=vr0dc8meaabaqaciGacaGaaeqabaqadeqadaaakeaaieaaceWF4bGbaebadaWgaaWcbaGaaGymaaqabaGccqGHsislceWF4bGbaebadaWgaaWcbaGaaGOmaaqabaaaaa@381E@ (see Equation 1 in the text) for probes from null genes from the comparison of Latin Square experiments 1 and 2 for **(a) **all approximately 500,000 probes on the array, **(b) **approximately 22,000 probes after RMA summation but in the absence of quantile-quantile normalization, and **(c) **after background correction, quantile-quantile normalization, and RMA summation. A small number of outlying data points are excluded from each panel. RMA, Robust Multichip Average.

Affymetrix microarrays have multiple 25-mer probes for each gene on the chip. In the Latin Square dataset, there are about 500,000 25-mer probes. These probes are organized into probesets that target about 22,000 genes. Because there are multiple probes in each probeset, we do not expect all the probes to act independently of one another. Nonetheless, in order to examine the distribution of variances on a microarray, it is informative to begin our analysis at the probe level. Figure 1a shows the pooled error (*σ*^2 ^from Equation 2) as a function of the mean difference (x¯1−x¯2
 MathType@MTEF@5@5@+=feaafiart1ev1aaatCvAUfeBSjuyZL2yd9gzLbvyNv2Caerbhv2BYDwAHbqedmvETj2BSbqee0evGueE0jxyaibaiKI8=vI8tuQ8FMI8Gi=hEeeu0xXdbba9frFj0=OqFfea0dXdd9vqai=hGuQ8kuc9pgc9s8qqaq=dirpe0xb9q8qiLsFr0=vr0=vr0dc8meaabaqaciGacaGaaeqabaqadeqadaaakeaaieaaceWF4bGbaebadaWgaaWcbaGaaGymaaqabaGccqGHsislceWF4bGbaebadaWgaaWcbaGaaGOmaaqabaaaaa@381E@ from Equation 1) of the approximately 500,000 probes from probesets that represent null (not spiked in) genes from the comparison between Latin Square experiments 1 and 2. In this case, there are three chips in each condition so n_1 _= n_2 _= 3. To make this figure consistent with the data shown in the rest of this report, all of the data from all arrays in Figure 1a were log_2 _transformed before calculation of x¯1−x¯2
 MathType@MTEF@5@5@+=feaafiart1ev1aaatCvAUfeBSjuyZL2yd9gzLbvyNv2Caerbhv2BYDwAHbqedmvETj2BSbqee0evGueE0jxyaibaiKI8=vI8tuQ8FMI8Gi=hEeeu0xXdbba9frFj0=OqFfea0dXdd9vqai=hGuQ8kuc9pgc9s8qqaq=dirpe0xb9q8qiLsFr0=vr0=vr0dc8meaabaqaciGacaGaaeqabaqadeqadaaakeaaieaaceWF4bGbaebadaWgaaWcbaGaaGymaaqabaGccqGHsislceWF4bGbaebadaWgaaWcbaGaaGOmaaqabaaaaa@381E@ and *σ*^2^. We would expect, based on previous literature, a relationship to exist between probe intensity and probe variance on microarrays [6]. We see in Figure 1a that such a relationship does in fact exist and that x¯1−x¯2
 MathType@MTEF@5@5@+=feaafiart1ev1aaatCvAUfeBSjuyZL2yd9gzLbvyNv2Caerbhv2BYDwAHbqedmvETj2BSbqee0evGueE0jxyaibaiKI8=vI8tuQ8FMI8Gi=hEeeu0xXdbba9frFj0=OqFfea0dXdd9vqai=hGuQ8kuc9pgc9s8qqaq=dirpe0xb9q8qiLsFr0=vr0=vr0dc8meaabaqaciGacaGaaeqabaqadeqadaaakeaaieaaceWF4bGbaebadaWgaaWcbaGaaGymaaqabaGccqGHsislceWF4bGbaebadaWgaaWcbaGaaGOmaaqabaaaaa@381E@ and *σ*^2 ^are not independent at the probe level.

We argue in our report that *σ*^2 ^can be thought of as approximately constant. This is clearly not true at the probe level in Figure 1a. Microarray analysis, however, is usually not performed directly at the probe level. For many microarray experiments, the desired analysis is at the gene level. A well studied problem in the analysis of Affymetrix arrays is how best to summarize the multiple probes in a probeset to produce a single value for each gene on each chip [7-10]. All of the probeset data in this report were generated with the log_2_-transformed Robust Multichip Average (RMA) summary statistic [8], which is a well regarded and robust measurement that has been shown to work well in a variety of conditions [11]. After transformation with the RMA statistic, our data can be represented as a single spreadsheet or matrix in which the columns represent experiments and the rows represent genes.

Figure 1b shows *σ*^2 ^as a function of x¯1−x¯2
 MathType@MTEF@5@5@+=feaafiart1ev1aaatCvAUfeBSjuyZL2yd9gzLbvyNv2Caerbhv2BYDwAHbqedmvETj2BSbqee0evGueE0jxyaibaiKI8=vI8tuQ8FMI8Gi=hEeeu0xXdbba9frFj0=OqFfea0dXdd9vqai=hGuQ8kuc9pgc9s8qqaq=dirpe0xb9q8qiLsFr0=vr0=vr0dc8meaabaqaciGacaGaaeqabaqadeqadaaakeaaieaaceWF4bGbaebadaWgaaWcbaGaaGymaaqabaGccqGHsislceWF4bGbaebadaWgaaWcbaGaaGOmaaqabaaaaa@381E@ for the approximately 22,000 probesets generated by the comparison of Latin Square experiments 1 and 2 after RMA summation. We note immediately that RMA summation suppresses the standard error. The values for probeset *σ*^2 ^in Figure 1b are on the order of 10 to 20 times smaller than the probe *σ*^2 ^observed in Figure 1a. In addition, we can tell by immediate inspection that the estimates of *σ*^2 ^in Figure 1b must contain errors because they are not symmetrical. The data in Figure 1 are from null (not spiked in) genes. The expected value of x¯1−x¯2
 MathType@MTEF@5@5@+=feaafiart1ev1aaatCvAUfeBSjuyZL2yd9gzLbvyNv2Caerbhv2BYDwAHbqedmvETj2BSbqee0evGueE0jxyaibaiKI8=vI8tuQ8FMI8Gi=hEeeu0xXdbba9frFj0=OqFfea0dXdd9vqai=hGuQ8kuc9pgc9s8qqaq=dirpe0xb9q8qiLsFr0=vr0=vr0dc8meaabaqaciGacaGaaeqabaqadeqadaaakeaaieaaceWF4bGbaebadaWgaaWcbaGaaGymaaqabaGccqGHsislceWF4bGbaebadaWgaaWcbaGaaGOmaaqabaaaaa@381E@ is therefore zero and there is no reason to believe that *σ*^2 ^should deviate from symmetry around zero. Clearly, in Figure 1b, however, there is a strong tendency for *σ*^2 ^to be larger when x¯1−x¯2
 MathType@MTEF@5@5@+=feaafiart1ev1aaatCvAUfeBSjuyZL2yd9gzLbvyNv2Caerbhv2BYDwAHbqedmvETj2BSbqee0evGueE0jxyaibaiKI8=vI8tuQ8FMI8Gi=hEeeu0xXdbba9frFj0=OqFfea0dXdd9vqai=hGuQ8kuc9pgc9s8qqaq=dirpe0xb9q8qiLsFr0=vr0=vr0dc8meaabaqaciGacaGaaeqabaqadeqadaaakeaaieaaceWF4bGbaebadaWgaaWcbaGaaGymaaqabaGccqGHsislceWF4bGbaebadaWgaaWcbaGaaGOmaaqabaaaaa@381E@ exceeds zero. This must be due to some systematic error in the underlying data. RMA summation is usually accompanied by quantile-quantile normalization [8], which is designed to correct for systematic errors in microarray data. Figure 1c shows the relationship between *σ*^2 ^and x¯1−x¯2
 MathType@MTEF@5@5@+=feaafiart1ev1aaatCvAUfeBSjuyZL2yd9gzLbvyNv2Caerbhv2BYDwAHbqedmvETj2BSbqee0evGueE0jxyaibaiKI8=vI8tuQ8FMI8Gi=hEeeu0xXdbba9frFj0=OqFfea0dXdd9vqai=hGuQ8kuc9pgc9s8qqaq=dirpe0xb9q8qiLsFr0=vr0=vr0dc8meaabaqaciGacaGaaeqabaqadeqadaaakeaaieaaceWF4bGbaebadaWgaaWcbaGaaGymaaqabaGccqGHsislceWF4bGbaebadaWgaaWcbaGaaGOmaaqabaaaaa@381E@ after both quantile-quantile normalization and RMA summation. We see that after quantile-quantile normalization, the standard error approaches a constant across the range of x¯1−x¯2
 MathType@MTEF@5@5@+=feaafiart1ev1aaatCvAUfeBSjuyZL2yd9gzLbvyNv2Caerbhv2BYDwAHbqedmvETj2BSbqee0evGueE0jxyaibaiKI8=vI8tuQ8FMI8Gi=hEeeu0xXdbba9frFj0=OqFfea0dXdd9vqai=hGuQ8kuc9pgc9s8qqaq=dirpe0xb9q8qiLsFr0=vr0=vr0dc8meaabaqaciGacaGaaeqabaqadeqadaaakeaaieaaceWF4bGbaebadaWgaaWcbaGaaGymaaqabaGccqGHsislceWF4bGbaebadaWgaaWcbaGaaGOmaaqabaaaaa@381E@ scores. In the following section we show that the deviations from a constant value of *σ*^2 ^that remain after normalization and RMA summation are likely to contain errors because, even on normalized data, test statistics work better if they assume that *σ*^2 ^is constant.

### The measured standard error either before or after quantile-quantile normalization is unreliable

In order to produce a reliable list of differentially expressed genes between two experimental conditions, we need a test statistic and an appropriate way to produce *P *values from that test statistic. It has recently become clear that the standard *t *test (Equations 1 and 2) has serious shortcomings as a test statistic for microarray data [11-13]. There has been a great deal of recent interest in test statistics that ignore or 'shrink' the variance of each individual gene. For example, a popular alternative to the standard *t *test is the cyber *t *test [12], which uses Bayesian statistics to weight the variance of each individual gene with the variance of other genes on the array with similar intensities (see Materials and methods, below). In addition to the cyber *t *test, we can follow Allison and coworkers [11] and describe a universe of possible test statistics with which to evaluate the null hypothesis that the expression of a given gene is the same in conditions 1 and 2:

x¯1−x¯2Bθ2+(1−B)σ2
 MathType@MTEF@5@5@+=feaafiart1ev1aaatCvAUfeBSjuyZL2yd9gzLbvyNv2Caerbhv2BYDwAHbqedmvETj2BSbqee0evGueE0jxyaibaiKI8=vI8tuQ8FMI8Gi=hEeeu0xXdbba9frFj0=OqFfea0dXdd9vqai=hGuQ8kuc9pgc9s8qqaq=dirpe0xb9q8qiLsFr0=vr0=vr0dc8meaabaqaciGacaGaaeqabaqadeqadaaakeaadaWcaaqaaGqaaiqa=HhagaqeamaaBaaaleaacaaIXaaabeaakiabgkHiTiqa=HhagaqeamaaBaaaleaacaaIYaaabeaaaOqaamaakaaabaGaa8NqaiabeI7aXnaaCaaaleqabaGaaGOmaaaakiabgUcaRiaacIcacaaIXaGaeyOeI0Iaa8NqaiaacMcaiiGacqGFdpWCdaahaaWcbeqaaiaaikdaaaaabeaaaaaaaa@430C@

Here, *σ*^2 ^is the estimate of standard error for each gene, as in the denominator for the *t *statistic in Equation 1. On the other hand, *θ*^2 ^is an estimate of the standard error of every gene on the array. We take as our *θ*^2 ^simply the average of all σ^2 ^values. That is, if there are *N *genes on the array, then:

θ2=∑i=1Nσi2N
 MathType@MTEF@5@5@+=feaafiart1ev1aaatCvAUfeBSjuyZL2yd9gzLbvyNv2Caerbhv2BYDwAHbqedmvETj2BSbqee0evGueE0jxyaibaiKI8=vI8tuQ8FMI8Gi=hEeeu0xXdbba9frFj0=OqFfea0dXdd9vqai=hGuQ8kuc9pgc9s8qqaq=dirpe0xb9q8qiLsFr0=vr0=vr0dc8meaabaqaciGacaGaaeqabaqadeqadaaakeaacqaH4oqCdaahaaWcbeqaaiaaikdaaaGccqGH9aqpdaaeWbqaamaalaaabaacciGae83Wdm3aa0baaSqaaGqaaiaa+LgaaeaacaaIYaaaaaGcbaGaa4NtaaaaaSqaaiaa+LgacqGH9aqpcaaIXaaabaGaa4NtaaqdcqGHris5aaaa@4123@

The shrinkage factor, B, can vary between 0 and 1 in Equation 3. When B = 0, Equation 3 reduces to the standard *t *test of Equation 1. When B = 1, the statistic essentially ignores the variance, in that it reduces to assigning a score based only on the average difference between the genes divided by a constant.

**Figure 2:**
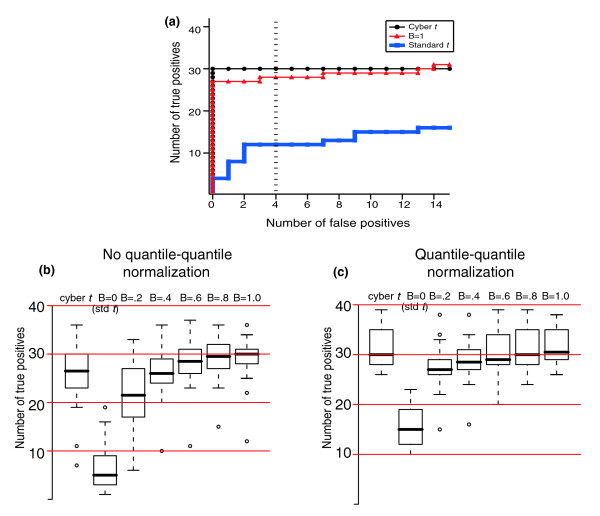
The performance of test statistics in ranking the Latin Square data. **(a) **ROC curves for Latin Square experiments 8 versus 9. **(b,c) **The number of true positives captured for all 14 2× Latin Square experiments at a threshold that also captured four false positives (dashed line in panel a) in the absence (panel b) and presence (panel c) of background correction and quantile-quantile normalization. B refers to the 'shrinkage factor' in Equation 3 (see text). For this and the following figures in the report, data were summarized with RMA before application of the test statistic. RMA, Robust Multichip Average; ROC, receiver operating characteristic.

The consequences of choosing different summary statistics are shown in Figure 2. A receiver operating characteristics (ROC) graph is shown in Figure 2a, in which we use different statistics to rank the most differentially expressed genes in Latin Square experiment 8 versus experiment 9. To generate an ROC curve for each statistic, we assign a score to each gene on the chip and sort the resulting list. For each gene in the sorted list we ask, if the threshold for significance were set to include only the genes with scores equal to or greater than the current gene, then how many true positives and false positives would be captured? An algorithm capable of perfectly separating true and false positives would generate a curve that would include a point in the upper left corner of Figure 2a, because there would exist a threshold cutoff in which all 42 spiked-in genes would be captured and all approximately 22,000 null genes would be excluded. We see in Figure 2a that the standard *t *test performs poorly whereas the cyber *t *test does well, as does the statistic defined by Equation 3 with B = 1.

To explore the effects of variance shrinkage and normalization on statistic performance across multiple Latin Square comparisons, we choose an arbitrary threshold; we consider how many true positives are captured by each statistic for a threshold cutoff that also captures four false positives (Figure 2a, dashed vertical line). The box plots in Figure 2 show this value for each statistic over the 14 Latin Square experiments in which the spiked-in ratios differ by a factor of two in the absence (Figure 2b) and presence (Figure 2c) of quantile-quantile normalization. We note that whether one uses Bayesian statistic to weigh the variance of each gene (as in the cyber *t *test) or shrinks the standard error according to Equation 3 (with B approaching 1), much better performance is achieved than with the standard *t *test, regardless of normalization schemes. This suggests that both before and after quantile-quantile normalization, the variance reported for each gene is unreliable.

**Figure 3:**
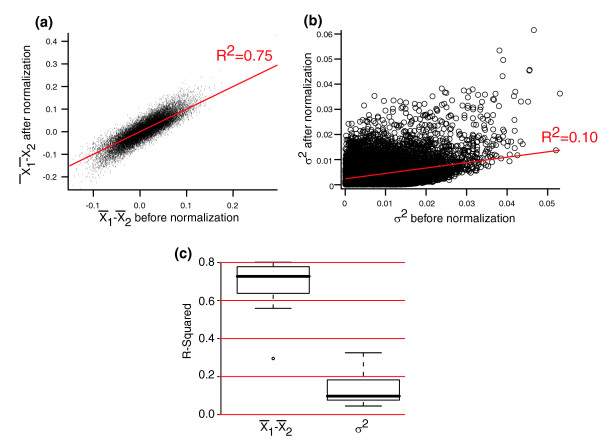
Estimates of standard error do not survive quantile-quantile normalization. **(a,b) **x¯1−x¯2
 MathType@MTEF@5@5@+=feaafiart1ev1aaatCvAUfeBSjuyZL2yd9gzLbvyNv2Caerbhv2BYDwAHbqedmvETj2BSbqee0evGueE0jxyaibaiKI8=vI8tuQ8FMI8Gi=hEeeu0xXdbba9frFj0=OqFfea0dXdd9vqai=hGuQ8kuc9pgc9s8qqaq=dirpe0xb9q8qiLsFr0=vr0=vr0dc8meaabaqaciGacaGaaeqabaqadeqadaaakeaaieaaceWF4bGbaebadaWgaaWcbaGaaGymaaqabaGccqGHsislceWF4bGbaebadaWgaaWcbaGaaGOmaaqabaaaaa@381E@ (panel a) and *σ*^2 ^(panel b) before and after background correction and quantile-quantile normalization for the comparison of the Latin Square experiments 1 and 2. Fits shown are to a linear regression. **(c) **Box plot showing the R^2 ^values from a linear fit for all 14 2× Latin Square comparisons.

In Figure 2, the B = 1 form of Equation 3 performs nearly the same in the absence (Figure 2b) and presence (Figure 2c) of quantile-quantile normalization. In contrast, the standard *t *test performs much better under quantile-quantile normalization (Figure 2c) than with un-normalized data (Figure 2b). This improvement must occur because either x¯1−x¯2
 MathType@MTEF@5@5@+=feaafiart1ev1aaatCvAUfeBSjuyZL2yd9gzLbvyNv2Caerbhv2BYDwAHbqedmvETj2BSbqee0evGueE0jxyaibaiKI8=vI8tuQ8FMI8Gi=hEeeu0xXdbba9frFj0=OqFfea0dXdd9vqai=hGuQ8kuc9pgc9s8qqaq=dirpe0xb9q8qiLsFr0=vr0=vr0dc8meaabaqaciGacaGaaeqabaqadeqadaaakeaaieaaceWF4bGbaebadaWgaaWcbaGaaGymaaqabaGccqGHsislceWF4bGbaebadaWgaaWcbaGaaGOmaaqabaaaaa@381E@ or *σ*^2^, or both, improve after normalization. Figure 3 shows the relationship between x¯1−x¯2
 MathType@MTEF@5@5@+=feaafiart1ev1aaatCvAUfeBSjuyZL2yd9gzLbvyNv2Caerbhv2BYDwAHbqedmvETj2BSbqee0evGueE0jxyaibaiKI8=vI8tuQ8FMI8Gi=hEeeu0xXdbba9frFj0=OqFfea0dXdd9vqai=hGuQ8kuc9pgc9s8qqaq=dirpe0xb9q8qiLsFr0=vr0=vr0dc8meaabaqaciGacaGaaeqabaqadeqadaaakeaaieaaceWF4bGbaebadaWgaaWcbaGaaGymaaqabaGccqGHsislceWF4bGbaebadaWgaaWcbaGaaGOmaaqabaaaaa@381E@ and *σ*^2 ^before (Figure 3a) and after (Figure 3b) quantile-quantile normalization for the comparison of experiments 1 and 2 in the Latin Square dataset. We see that x¯1−x¯2
 MathType@MTEF@5@5@+=feaafiart1ev1aaatCvAUfeBSjuyZL2yd9gzLbvyNv2Caerbhv2BYDwAHbqedmvETj2BSbqee0evGueE0jxyaibaiKI8=vI8tuQ8FMI8Gi=hEeeu0xXdbba9frFj0=OqFfea0dXdd9vqai=hGuQ8kuc9pgc9s8qqaq=dirpe0xb9q8qiLsFr0=vr0=vr0dc8meaabaqaciGacaGaaeqabaqadeqadaaakeaaieaaceWF4bGbaebadaWgaaWcbaGaaGymaaqabaGccqGHsislceWF4bGbaebadaWgaaWcbaGaaGOmaaqabaaaaa@381E@ is perturbed much less than *σ*^2 ^by normalization. The fact that the standard *t *test improves after normalization (Figure 2), however, suggests that the *σ*^2 ^values after normalization are more appropriate. This is something of a paradox. How can a transformation that discards about 90% of the original estimates for *σ*^2 ^improve performance? We argue that the resolution to this apparent paradox is that the original estimates of *σ*^2 ^after RMA summation are highly unreliable. Quantile-quantile normalization replaces the original estimates of *σ*^2 ^with values that approach a constant (Figure 1c). This improves the performance of the standard *t *test (Figure 2). That is, quantile-quantile normalization suppresses the original measured variance and therefore allows the standard *t *test to move closer to the performance of algorithms, such as cyber *t *test and the B = 1 form of Equation 1, that suppress the importance of the original variance regardless of normalization schemes.

### Different analysis schemes yield very different distributions of *P *values

We have argued that quantile-quantile normalization is effective in part because it replaces the unreliable estimates of *σ*^2 ^with a distribution that approaches a constant (Figures 1 and 3) and that, furthermore, test statistics appear to work better when they assume that *σ*^2 ^approaches a constant (Figure 2). We now turn to the issue of how we can utilize this assumption of constant standard error to produce more accurate estimates of *P *values.

**Figure 4:**
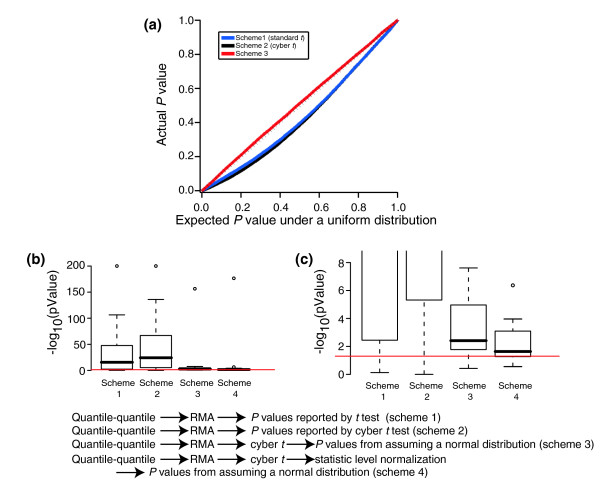
Actual versus expected *P *values under uniform distribution for null genes of Latin Square 2× comparisons. **(a) **Actual versus expected *P *values for the comparison of experiment 10 versus 11. Black dashes indicate the y = x diagonal. **(b) **Box plots showing the results of the Kolmogorov-Smirnov test for each of the 14 Latin Square 2× comparisons under the null hypothesis that the observed distribution of *P *values was the same as a uniform distribution of *P *values. The red line is the *P *= 0.05 level. **(c) **Same data as in panel b but with a magnified y-axis.

If the assumptions of normality, equal variance, and independence were met, then we would of course expect the standard *t *test in Equation 1 to follow a *t *distribution with appropriate degrees of freedom for null genes. If any of these assumptions are violated, however, then the distribution of standard *t *scores may not follow a *t *distribution. We can examine how well these assumptions are met for the standard *t *test by using the *t *distribution to produce *P *values for null genes. If all the assumptions are met, then the *P *values produced from the *t *distribution should follow a uniform distribution. Figure 4a (blue lines) shows that the *P *values produced by the *t *distribution for the standard *t *test (with four degrees of freedom, because n_10 _= n_11 _= 3) compared with the expected *P *values under a uniform distribution for the comparison of Latin Square experiments 10 versus 11 after RMA summation and quantile-quantile normalization. We see that the actual distribution of *P *values produced by the standard *t *test deviates considerably from the expected *P *values. Clearly, one or more of the assumptions of the standard *t *test is violated in this case.

Given the poor performance of the standard *t *test in ranking differentially expressed genes (Figure 2), it is perhaps not surprising that the *P *values generated by the standard *t *test fall so far from uniform. Does the cyber *t *test, which clearly outperforms the standard *t *test in ranking differentially expressed genes (Figure 2), produce *P *values closer to a uniform distribution? Rather than determining *σ*^2 ^independently for each gene, the cyber *t *test uses Bayesian statistics to weigh the variance of each gene by the variance of genes on the array with similar intensities. Because the estimate for the variance of each gene is not independent, the authors of the cyber *t *test do not expect the cyber *t *test to follow a simple *t *distribution with n - 2 degrees of freedom. Indeed, the *P *values reported by the R implementation of the cyber *t *test that we used are generated with an assumption of 22 degrees of freedom, given three experiments in each condition and the default parameters (see Materials and methods, below). Figure 4a (black lines) presents the *P *values reported by the R implementation of the cyber *t *test. We see that, despite the correction for lack of independence by increasing the number of degrees of freedom, the *P *values reported by the cyber *t *test are also poorly described by a uniform distribution.

If the cyber *t *test does not appear to follow a *t *distribution, then can we find a more appropriate distribution that it does follow? In Figure 1c, we have seen that *σ*^2 ^approaches a constant for null genes after RMA summation and quantile-quantile normalization. The cyber *t *test estimates the prior variance of each gene as a function of that gene's intensity. After RMA summation and quantile-quantile normalization, that prior variance should be close to constant. Because in the Latin Square dataset we have small sample sizes, the Bayesian cyber *t *estimate gives a good deal of weight to the prior variance, and therefore the cyber *t *estimate of variance for each gene will also approach a constant. As a distribution approaches x¯1−x¯2
 MathType@MTEF@5@5@+=feaafiart1ev1aaatCvAUfeBSjuyZL2yd9gzLbvyNv2Caerbhv2BYDwAHbqedmvETj2BSbqee0evGueE0jxyaibaiKI8=vI8tuQ8FMI8Gi=hEeeu0xXdbba9frFj0=OqFfea0dXdd9vqai=hGuQ8kuc9pgc9s8qqaq=dirpe0xb9q8qiLsFr0=vr0=vr0dc8meaabaqaciGacaGaaeqabaqadeqadaaakeaaieaaceWF4bGbaebadaWgaaWcbaGaaGymaaqabaGccqGHsislceWF4bGbaebadaWgaaWcbaGaaGOmaaqabaaaaa@381E@ divided by a constant, it will become normally distributed. We might anticipate, therefore, that the distribution of all cyber *t *scores should approach a normal distribution.

We can check the validity of the above line of reasoning by generating *P *values for the cyber *t *scores under the assumption that they are normally distributed. For the comparison of null genes between Latin Square experiments 10 and 11, we calculate the mean (cyberT¯
 MathType@MTEF@5@5@+=feaafiart1ev1aaatCvAUfeBSjuyZL2yd9gzLbvyNv2Caerbhv2BYDwAHbqedmvETj2BSbqee0evGueE0jxyaibaiKI8=vI8tuQ8FMI8Gi=hEeeu0xXdbba9frFj0=OqFfea0dXdd9vqai=hGuQ8kuc9pgc9s8qqaq=dirpe0xb9q8qiLsFr0=vr0=vr0dc8meaabaqaciGacaGaaeqabaqadeqadaaakeaadaqdaaqaaGqaaiaa=ngacaWF5bGaa8Nyaiaa=vgacaWFYbGaa8hvaaaaaaa@38B6@) and standard deviation (*σ*_cyberT_) of all the cyber *t *scores. We can then easily calculate the *P *value from the cumulative distribution function (cdf) of the standard normal distribution for each cyberT score as follows:

p(cyberT)=2*cdf(|cyberT−cyberT¯|σcyberT)
 MathType@MTEF@5@5@+=feaafiart1ev1aaatCvAUfeBSjuyZL2yd9gzLbvyNv2Caerbhv2BYDwAHbqedmvETj2BSbqee0evGueE0jxyaibaiKI8=vI8tuQ8FMI8Gi=hEeeu0xXdbba9frFj0=OqFfea0dXdd9vqai=hGuQ8kuc9pgc9s8qqaq=dirpe0xb9q8qiLsFr0=vr0=vr0dc8meaabaqaciGacaGaaeqabaqadeqadaaakeaaieaacaWFWbGaaiikaiaa=ngacaWF5bGaa8Nyaiaa=vgacaWFYbGaa8hvaiaacMcacqGH9aqpcaaIYaGaaiOkaiaa=ngacaWFKbGaa8NzaiaacIcadaWcaaqaaiaacYhacaWFJbGaa8xEaiaa=jgacaWFLbGaa8NCaiaa=rfacqGHsisldaqdaaqaaiaa=ngacaWF5bGaa8Nyaiaa=vgacaWFYbGaa8hvaaaacaGG8baabaGaeq4Wdm3aaSbaaSqaaiaa=ngacaWF5bGaa8Nyaiaa=vgacaWFYbGaa8hvaaqabaaaaOGaaiykaaaa@56BC@

Figure 4a (red line) shows that the *P *values produced by the normal distribution of Equation 4 fall very close to a uniform distribution. This provides strong evidence that our assertion that the cyber *t *estimate of *σ*^2 ^is approximately constant is reasonable. For the rest of this report, we refer to the method of generating *P *values from the cyber *t *test by assuming a normal distribution as 'cyber-*t*-Normal'. We emphasize that there are two differences between *P *values produced by cyber-*t*-Normal and the *P *values reported by the cyber *t *test. One is that we assume a normal distribution rather than a *t *distribution. The other is that we calculate the *P *value for each gene by comparison with a distribution of all genes on the array. That is, we assume that all the genes on the array follow a single distribution whereas the *P *values produced by the cyber *t *test are generated under the assumption that each gene follows its own independent distribution based on the Bayesian estimate of *σ*^2 ^for that gene.

How close are the *P *values produced by the cyber-*t*-Normal scheme to a uniform distribution? We can use the Kolmogorov-Smirnov test to evaluate the null hypothesis that the distribution of *P *values from each statistic is identical to the uniform distribution of *P *values. The Kolmogorov-Smirnov test is a nonparametric test and can therefore suffer from low power. On the other hand, we are using the test to evaluate a distribution with over 22,000 data points, and so we are confident that even small deviations from our assumptions will produce small *P *values. Figure 4b,c shows the -log_10 _of the *P *value of the Kolmogorov-Smirnov test for all 14 possible 2× Latin Square comparisons. We see that, although there is considerable variability across all 14 pairs of experiments, *P *values produced by the cyber-*t*-Normal method are a good deal closer to uniform than *P *values produced by either the standard *t *or cyber *t *methods.

### Imperfect normalization contributes to deviations from a perfectly normal distribution

**Figure 5:**
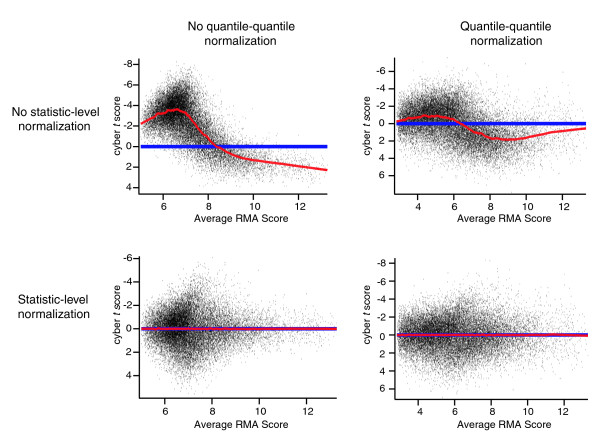
Fitting data to a local regression removes systematic variations present after quantile-quantile normalization. Shown is a comparison of cyber *t *scores for the null genes of the Latin Square comparison of experiment 8 versus 9 in the presence and absence of quantile-quantile and statistics level normalization (see text). Red lines are Lowess regression lines with a window size of 1,000.

The red lines in Figure 4b,c represent a *P *value of 0.05 for the null hypothesis that a statistic produces *P *values that are uniform. Figure 4c contains the same data as Figure 4b with a magnified scale. We see that even though the cyber-*t*-Normal method produces *P *values that are a good deal closer to uniform than the other methods, there is still significant deviation from a perfectly uniform distribution. One possible explanation for this deviation is imperfect normalization from the quantile-quantile procedure. The top panels in Figure 5 show cyber *t *scores after RMA summation in the presence (top right panel) and absence (top left panel) of quantile-quantile normalization for the null genes for a comparison of Latin Square experiments 8 and 9. We see that even after quantile-quantile normalization, there remain systematic differences in the null genes (top right panel). Such systematic differences even after normalization have been observed in other datasets [13-15]. To correct for these differences, we can perform an additional normalization, which we call 'statistics-level normalization'. To do this, we simply fit a local (Loess) regression line to the data in the top panels of Figure 5 with a window size of 1,000 data points. We then subtract from each gene the value for that gene from the Loess regression line. The results of this subtraction are shown in the bottom panels of Figure 5. We see in Figure 4b,c that when we perform this additional normalization, the *P *values produced by cyber-*t*-Normal become slightly closer to uniform. For the rest of the report, we refer to the calculation of *P *values by cyber-*t*-Normal after RMA summation, quantile-quantile normalization, and statistic-level normalization as 'scheme 4'.

### Cross-hybridization also contributes to deviations from a perfectly normal distribution

Another possible cause of deviations from the normal distribution in Figure 4 is 'off-target' or cross-hybridization. We might expect that some probe sets respond to changes in genes other than those that they were designed to detect. If genes that are annotated as null are in fact responding to changes in spiked-in genes, this would cause *P *values to be smaller than expected under a uniform distribution. We can examine the effect of cross-hybridization by taking advantage of the experimental design of the Latin Square dataset. For each of the 91 possible pairs of experiments in the Latin Square dataset, we can compute the average difference between spike-in concentrations. That is, if the spike-in concentrations for the 42 genes in experiment X are ([X_1_], [X_2_], [X_3_] ... [X_42_]) and for experiment Y are ([Y_1_], [Y_2_], [Y_3_] ... [Y_42_]), then we define the average difference in concentration as follows:

∑i=142|Xi−Yi|42
 MathType@MTEF@5@5@+=feaafiart1ev1aaatCvAUfeBSjuyZL2yd9gzLbvyNv2Caerbhv2BYDwAHbqedmvETj2BSbqee0evGueE0jxyaibaiKI8=vI8tuQ8FMI8Gi=hEeeu0xXdbba9frFj0=OqFfea0dXdd9vqai=hGuQ8kuc9pgc9s8qqaq=dirpe0xb9q8qiLsFr0=vr0=vr0dc8meaabaqaciGacaGaaeqabaqadeqadaaakeaadaWcaaqaamaaqahabaWaaqWaaeaaieaacaWFybWaaSbaaSqaaiaa=LgaaeqaaOGaeyOeI0Iaa8xwamaaBaaaleaacaWFPbaabeaaaOGaay5bSlaawIa7aaWcbaGaa8xAaiabg2da9iaaigdaaeaacaaI0aGaaGOmaaqdcqGHris5aaGcbaGaaGinaiaaikdaaaaaaa@4333@

**Figure 6:**
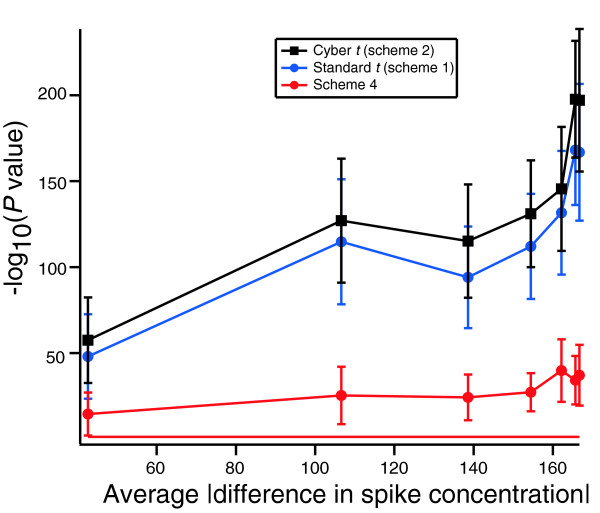
Cross-hybridization distorts *P *values for null genes in the Latin Square dataset. Shown are the results of the Kolmogorov-Smirnov test for the null genes for all 91 Latin Square comparisons as a function of the average difference in spike concentration (see text). The null hypothesis for the Kolmogorov-Smirnov test is that the observed *P *values are identical to a uniform distribution. Error bars are standard errors. The red line is the *P *= 0.05 level.

Figure 6 shows the -log_10 _(pValues) from the Kolmogorov-Smirnov test as a function of this average difference in spike-in concentration. As in Figure 4, the Kolmogorov-Smirnov test evaluates the null hypothesis that the distribution of *P *values produced by each statistic follows a uniform distribution. As we go from left to right on the x axis, we find experiments in which the arrays were exposed to greater differences in RNA concentrations. The data in this figure were constructed from a dataset containing only null genes. Despite the fact that the spike-in genes are removed from this dataset, we see an increase in the deviation from a uniform distribution as spike-in concentration increases. This must be due to nonspecific hybridization. That is, probes that target null genes are responding to changes in the spiked-in genes. Because even in the 2× comparisons, the chips in the two conditions are exposed to some differences in RNA, we can explain some of the deviations from the normal distribution in Figure 4 by cross-hybridization.

### Experiments consisting of technical replicates are closer to a normal distribution

Technical replicates consist of arrays that have been exposed to identical RNA. Every gene within a comparison of technical replicates is therefore a null gene. If some of the deviation from a uniform distribution in Figure 4 were caused by cross-hybridization, then we would anticipate that experiments consisting entirely of technical replicates would be closer to a uniform distribution. The sample sizes in the Latin Square experiment shown in Figure 4 are *n *= 3 for each condition, however, which does not allow for comparison within an experimental condition by either the cyber *t *or standard *t *test. Fortunately, a dataset with six technical replicates has been published [16]. This dataset, which was designed to measure the effect of different RNA amplification schemes, consists of six technical replicates in each of four distinct groups for a total of 24 arrays. Within each of the four groups, there are 10 possible ways to split the six technical replicates into two groups of three. There are therefore a total of 40 distinct comparisons of technical replicates with n_1 _= n_2 _= 3 within the 24 arrays of this dataset.

**Figure 7:**
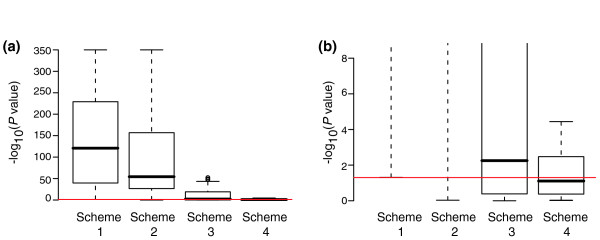
Actual versus expected *P *values for a technical replicate dataset. Shown are results of the Kolmogorov-Smirnov test for all 40 possible *n *= 3 versus *n *= 3 combinations of the technical replicates from the dataset of Cope and coworkers [16]. The null hypothesis for the Kolmogorov-Smirnov test is that the observed *P *values are identical to a uniform distribution. The red line is the *P *= 0.05 level. **(a,b) **The same data are shown in both panels but panel b has a magnified y-axis.

For each of these 40 possible *n *= 3 versus *n *= 3 comparisons of technical replicates, we used the Kolmogorov-Smirnov test to evaluate the null hypothesis that the *P *values produced by various schemes were identical to the uniform distribution of *P *values. The box plots in Figure 7 show the results of this calculation. Figure 7b is identical to Figure 7a except the y axis has been magnified. We see that for more than half of the 40 comparisons under 'scheme 4' there is no statistical difference between the generated *P *values and the uniform distribution at a *P *value cutoff of 0.05. The fact that the distribution of *P *values produced by 'scheme 4' for these technical replicates is closer to a uniform distribution than for the null genes of the 2× Latin Square experiments in Figure 4 suggests that some of the deviation from a uniform distribution in Figure 4 is caused by cross-hybridization.

### 'Scheme 4' should be conservative in real experiments

The graphs in Figures 4 to 7 were created using data from only null genes, which we know are not differentially expressed. In 'real' experiments, of course, we will have a mixture of null and not-null genes and we will not know which genes are null and which are differentially expressed. When we compare genes in two conditions, we assume that null genes will follow a normal distribution of scores whereas genes that are not null will not follow this same distribution. Because the majority of genes are probably null, the overall distribution of scores from a test statistic will largely reflect null genes. We measure the significance of genes as deviations from this background distribution of presumably null genes. Of course, not all of the genes will be null, and we will therefore not be able to measure cyberTNulls¯
 MathType@MTEF@5@5@+=feaafiart1ev1aaatCvAUfeBSjuyZL2yd9gzLbvyNv2Caerbhv2BYDwAHbqedmvETj2BSbqee0evGueE0jxyaibaiKI8=vI8tuQ8FMI8Gi=hEeeu0xXdbba9frFj0=OqFfea0dXdd9vqai=hGuQ8kuc9pgc9s8qqaq=dirpe0xb9q8qiLsFr0=vr0=vr0dc8meaabaqaciGacaGaaeqabaqadeqadaaakeaadaqdaaqaaGqaaiaa=ngacaWF5bGaa8Nyaiaa=vgacaWFYbGaa8hvaiaa=5eacaWF1bGaa8hBaiaa=XgacaWFZbaaaaaa@3D49@ and *σ*_cyberTNulls _(the average and standard deviation of cyber *t *scores from null genes) but only cyberTAll¯
 MathType@MTEF@5@5@+=feaafiart1ev1aaatCvAUfeBSjuyZL2yd9gzLbvyNv2Caerbhv2BYDwAHbqedmvETj2BSbqee0evGueE0jxyaibaiKI8=vI8tuQ8FMI8Gi=hEeeu0xXdbba9frFj0=OqFfea0dXdd9vqai=hGuQ8kuc9pgc9s8qqaq=dirpe0xb9q8qiLsFr0=vr0=vr0dc8meaabaqaciGacaGaaeqabaqadeqadaaakeaadaqdaaqaaGqaaiaa=ngacaWF5bGaa8Nyaiaa=vgacaWFYbGaa8hvaiaa=feacaWFSbGaa8hBaaaaaaa@3B52@ and *σ*_cyberTAll_, which we define as the observed mean and standard deviation of cyber *t *scores for all genes.

We would still expect, however, the number of upregulated genes to be approximately equal to the number of downregulated genes. We expect, therefore, that:

cyberTNulls¯≈cyberTAll¯≈0
 MathType@MTEF@5@5@+=feaafiart1ev1aaatCvAUfeBSjuyZL2yd9gzLbvyNv2Caerbhv2BYDwAHbqedmvETj2BSbqee0evGueE0jxyaibaiKI8=vI8tuQ8FMI8Gi=hEeeu0xXdbba9frFj0=OqFfea0dXdd9vqai=hGuQ8kuc9pgc9s8qqaq=dirpe0xb9q8qiLsFr0=vr0=vr0dc8meaabaqaciGacaGaaeqabaqadeqadaaakeaadaqdaaqaaGqaaiaa=ngacaWF5bGaa8Nyaiaa=vgacaWFYbGaa8hvaiaa=5eacaWF1bGaa8hBaiaa=XgacaWFZbaaaiabgIKi7oaanaaabaGaa83yaiaa=LhacaWFIbGaa8xzaiaa=jhacaWFubGaa8xqaiaa=XgacaWFSbaaaiabgIKi7kaaicdaaaa@4981@

Moreover, cyber *t *scores will be higher for not-null genes than for null genes, and we therefore expect:

*σ*_cyberTAll _> *σ*_cyberTNulls_

Estimates of *P *values generated by Equation 4 with cyberTAll¯
 MathType@MTEF@5@5@+=feaafiart1ev1aaatCvAUfeBSjuyZL2yd9gzLbvyNv2Caerbhv2BYDwAHbqedmvETj2BSbqee0evGueE0jxyaibaiKI8=vI8tuQ8FMI8Gi=hEeeu0xXdbba9frFj0=OqFfea0dXdd9vqai=hGuQ8kuc9pgc9s8qqaq=dirpe0xb9q8qiLsFr0=vr0=vr0dc8meaabaqaciGacaGaaeqabaqadeqadaaakeaadaqdaaqaaGqaaiaa=ngacaWF5bGaa8Nyaiaa=vgacaWFYbGaa8hvaiaa=feacaWFSbGaa8hBaaaaaaa@3B52@ and *σ*_cyberTAll _will therefore tend to be larger than *P *values that would be calculated with cyberTNull¯
 MathType@MTEF@5@5@+=feaafiart1ev1aaatCvAUfeBSjuyZL2yd9gzLbvyNv2Caerbhv2BYDwAHbqedmvETj2BSbqee0evGueE0jxyaibaiKI8=vI8tuQ8FMI8Gi=hEeeu0xXdbba9frFj0=OqFfea0dXdd9vqai=hGuQ8kuc9pgc9s8qqaq=dirpe0xb9q8qiLsFr0=vr0=vr0dc8meaabaqaciGacaGaaeqabaqadeqadaaakeaadaqdaaqaaGqaaiaa=ngacaWF5bGaa8Nyaiaa=vgacaWFYbGaa8hvaiaa=5eacaWF1bGaa8hBaiaa=Xgaaaaaaa@3C55@ and *σ*_cyberTNull_s from only the null genes. As more and more genes are differentially expressed between two samples, conclusions based on the *P *values generated by Equation 4 should therefore become more conservative.

### Scheme 4 has attractive sensitivity and specificity when controlling false discovery rate

In order to compile a list of genes that are differentially expressed between conditions, one requires not only a set of *P *values but also some way to set a significance threshold controlling for family-wise error rate or FDR. There are a large number of reasonable choices that one could make in determining a threshold for significance [3,4,11,17]. In this report, we choose to set a threshold for significance using the Benjamini and Hochberg algorithm [18], which is a simple and popular method for controlling FDR.

**Figure 8:**
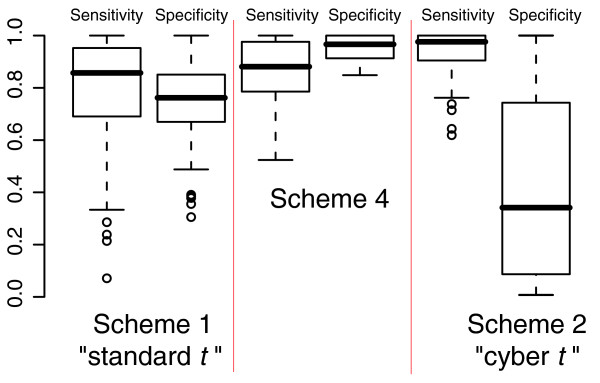
Sensitivity and specificity of different statistics for the Latin Square dataset. Sensitivity and specificity using the Benjamini and Hochberg algorithm to control false discovery rate at 10% using the *P *values supplied by the various schemes for all 91 possible pair-wise comparisons in the Latin Square dataset.

Figure 8 shows sensitivity and specificity for all 91 possible pair-wise comparisons in the Latin Square dataset at a FDR of 10%, as calculated using the Benjamini and Hochberg metric. We define sensitivity as the number of true positives recovered at the 10% FDR threshold divided by the total number of true positives in the Latin Square dataset. We define specificity as the number of true positives recovered at this threshold divided by the total number of genes recovered. At a 10% FDR, we expect a specificity of 0.9 or greater. We see that the *P *values generated by scheme 4 lead to appropriate balancing of sensitivity and specificity. For nearly all of the 91 comparisons, scheme 4 provides control of FDR at greater specificity than the expected 0.9, while maintaining an overall median sensitivity of about 0.9. In contrast, the *P *values generated using the standard *t *test and cyber *t *test lead to specificity that is considerably worse than the predicted FDR. We conclude that, at least for the Latin Square dataset, Benjamini and Hochberg control of FDR fails under standard *t *and cyber *t *but succeeds under scheme 4. These findings suggest that the *P *values produced by scheme 4 can lead to more appropriate cutoffs for gene lists than either the standard *t *or cyber *t *tests.

### On biologic replicates, scheme 4 yields conservative, but reasonable, estimates of significant genes

To assess the performance of scheme 4 on real, as opposed to spike-in data, we here present a previously unpublished dataset involving isogenic biologic replicates of untransformed mouse cell lines derived from a single individual. In these experiments, two cell lines, myeloid (MY) and embryoid blast (EB), were exposed for 60 min to either dimethyl sulfoxide (DMSO) along or DMSO plus the chemotherapeutic agent etoposide at 50 μmol/l. Cells were allowed to recover for either 4 hours or 24 hours. Five biological replicates (distinct plates of cells) in each condition were hybridized to the Mouse430_2 chip for a total of 40 experiments (two time points × two experimental conditions × two tissue types × five biologic replicates). For each time point at each tissue type, we consider how many genes are differentially expressed when comparing the cells exposed to drug with control cells. Table 1 shows the number of differentially expressed genes at a 10% FDR, as calculated in four different ways. For the standard *t *test, cyber *t *statistic, and scheme 4, we fed the *P *values generated by these tests into the Benjamini and Hochberg [18] FDR algorithm. For the significance analysis of microarrays (SAM) statistic [1], we used the implementation of SAM provided by TIGR mev (see Materials and methods, below). As might be expected based on the results from the Latin Square control dataset (Figure 5), we see in Table 1 that the *P *values from scheme 4 lead to a much more conservative estimate of significance than do the *P *values from cyber *t *test or the standard *t *test.

**Table 1:** Number of genes called significant at 10% false discovery rate on isogenic biologic replicates

Statistic	Embryoid blast		Myeloid	
	
	4 hours	24 hours	4 hours	24 hours
Standard *t *(scheme 1)	32	8,038	7,154	331
Cyber *t *(scheme 2)	1,288	9,769	10,349	4,464
Scheme 4	90	87	268	38
SAM	4,954	14,239	16,644	9,392

Each entry in this table is the number of significant genes when comparing treatment (DMSO + drug) with control (just DMSO) for a cell type (embryoid blast or myeloid) and a time point (4 or 24 hours). All data were subject to quantile-quantile normalization and summarized using RMA. The Benjamini and Hochberg algorithm was used on *P *values generated by the standard *t *test, cyber *t*, and scheme 4 to calculate false discovery rate. The values for SAM were generated by the TIGR Multiple Array Viewer (see Materials and methods) with 100 permutations and the default parameters. DMSO, dimethyl sulfoxide; RMA, Robust Multichip Average; SAM, significance analysis of microarrays.

**Figure 9:**
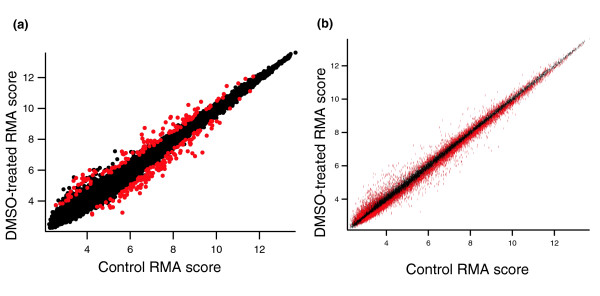
Positives at 10% FDR identified by scheme 4 and the standard *t *test. Shown are the averages of RMA scores across the five replicates comparing baseline (DMSO only) and experiment (DMSO + drug) for myeloid cells 4 hours after treatment. The same data are shown in both panels. **(a,b) **Red symbols show genes called significant in Table 1 from (panel b) the standard *t *test (scheme 1) and (panel a) under scheme 4 at a 10% false discovery rate (FDR), as calculated using the Benjamini and Hochberg algorithm.

How reasonable are the various predictions of differentially expressed genes shown in Table 1? Of course, because this is not a 'spike in' dataset, we do not know how many genes were truly differentially expressed. Nonetheless, we can still make some assessment of how the various algorithms perform. Figure 9 shows the average RMA score in treatment versus control for myeloid (MY) samples at 4 hours. The red symbols show the genes marked significant at 10% FDR under the standard *t *test (Figure 9b) and scheme 4 (Figure 9a). We note that the Pearson *r*^2 ^correlation between baseline and experiment averages in Figure 9 is 0.991. Given the subtle sources of noise in a microarray experiment such as cross-hybridization (Figure 5) [19] and the tight correlation between baseline and experiment samples, our findings of 7,154 differentially expressed genes through the standard *t *route in Table 1 seems unreasonable, as does the 10,349 genes found through *P *values reported by cyber t and the 16,644 genes found significant through the SAM analysis. We also note that in all four experimental conditions, there were no gross morphologic changes or obvious differences in growth between drug exposed and control groups (data not shown). If there really were many thousands of genes differentially expressed between drug and control groups, then one would expect to see large differences in the appearance and the behavior of the cells. The lack of such differences reinforces our argument that the more modest number of genes predicted to be differentially expressed by scheme 4 seems more reasonable than the results produced using other methods.

Although we have argued that the scheme 4 route to FDR should be conservative, given the tight correlation shown in Figure 9, it seems possible that we have over-estimated the number of genes that are truly differentially expressed. Is an assertion that there are in fact no differentially expressed genes in these experiments correct? We can take advantage of our experimental design to rule out that possibility. Of the 90 genes that are significant by scheme 4 based FDR between treatment and control (Table 1) for EB samples at 4 hours, 15 are also differentially expressed in the 87 found significant by scheme 4 in the EB samples at 24 hours. We can use the hypergeometric distribution to reject the null hypothesis that the genes found to be significant in EB samples at 4 hours are unrelated to the genes found to be significant in EB samples at 24 hours with *P *< 10^-25^. A significant fraction of the genes found to be significant with scheme 4 are therefore reproducibly differentially expressed across the 4-hour and 24-hour time points.

Likewise, of the 38 genes found to be significant by scheme 4 for MY samples at 24 hours, 15 are also differentially expressed in the 268 genes found to be significant by scheme 4 in the MY samples at 4 hours (*P *< 10^-24^). We have some evidence, therefore, that the scheme 4 route was not inappropriately anticonservative in this analysis. That is, at least some of the genes described by scheme 4 were indeed differentially expressed.

## Conclusion

In this paper we argue that microarray statistics work best when the estimate of standard error from each gene on the array is ignored or suppressed. We are not the first group to suggest that estimates of variance from individual genes are unreliable. Previous studies have noted improved statistics when a constant is added to the variance [1,11] or weighted by the variance from neighboring genes [12]. We argue that if the variance from each gene is truly unknown, then it makes sense to consider all of the genes on the array as arising from a single, normal distribution. We have demonstrated that this assumption of a single normal distribution of all genes comes much closer to producing a uniform distribution of *P *values than does production of *P *values from the *t *distribution (Figures 4 and 7).

It is not immediately clear why algorithms, such as the standard *t *test, that attempt to estimate the standard error of each individual gene perform so poorly. Difficulties in accurately estimating the variance of each individual gene may arise because of the modest sample sizes in typical microarray experiments. It has also been shown that the normalization process may distort the variance of genes [20,21]. We have seen, however, that the standard *t *test does much better on quantile-quantile normalized data than on non-normalized data (Figure 2), even though only about 10% of the original estimate of standard error survives normalization (Figure 3). The distortion of variance by normalization is apparently helpful. We argue that it is helpful because it replaces the original estimate of standard error for each gene with a distribution that approaches a small constant (Figure 1). This is consistent with the true variance for each gene being unknown regardless of normalization procedures.

The cyber *t *test uses Bayesian statistics to weigh the variance of each gene by the variance of genes with similar intensities on the array (see Materials and methods, below). As the experimental sample size increases, the weight given to the measured variance is increased while the weight given to the variance shared among similar genes is decreased. At large sample sizes, the performance of the cyber *t *test will therefore approach the performance of the standard *t *test. This behavior of the cyber *t *test is appropriate if the measured variance approaches the true variance as sample size increases. If, however, there are other factors at work in addition to small sample size that cause the measured variance to be unreliable, then the performance of the cyber *t *test may degrade as the sample size increases and the weight assigned to the background variance is therefore diminished. There is an urgent need for control datasets with larger sample sizes to determine whether the unreliability of the measured variance is primarily a function of small sample size or is somehow being caused by other aspects of microarray technology.

A recent controversy in the microarray literature has centered directly on the assumption of the uniform distribution of null *P *values. In analyzing a spike in dataset, Choe and coworkers [13] found that predicted FDRs from the SAM [1] algorithm appeared to be greatly anticonservative when compared with actual FDRs. In response, Dabney and Storey [5] noted that the anticonservative behavior of the SAM algorithm could be explained by the non-uniform distribution of *P *values among the non-spiked-in genes. In the Choe dataset, non-spiked-in genes had a surprising tendency to have *P *values too close to zero. Dabney and Storey argued that this non-uniform distribution was caused by errors in the experimental design of the spike-in dataset, a charge that was echoed somewhat by a second reanalysis of the Choe dataset [22]. These charges have been vigorously disputed by the authors of the Choe dataset, who argue that the non-uniform distribution of *P *values may be a common feature of microarray data [14,15].

Our study lends support to the arguments presented by Choe and coworkers. There are only 42 genes spiked in to the Latin Square dataset, but even this modest number of genes can produce detectable distortions in the distribution of *P *values among null genes (Figure 6). Given that the Choe dataset includes more than a thousand spiked-in genes, it is not surprising that the null genes in the Choe dataset have profoundly distorted *P *values. Moreover, the original analysis of the Choe dataset used cyber *t *[13], whereas the reanalysis used a standard *t *test [5]. We have shown that both of these tests can distort the distribution of null *P *values (Figure 4 and 7). In their reports [13,15], Choe and coworkers suggest multiple normalization steps as a way to avoid bias in the test statistics. We find that a second normalization step does make a small difference in producing uniform *P *values (Figures 4 and 7). We argue, however, that a larger difference can be made by finding a more appropriate distribution of microarray scores than the *t *distribution.

A problem with all microarray statistics papers is that they are dependent on the datasets analyzed. It is a constant worry that the assumptions made with regard to one dataset will not apply to new datasets in the future, that is to say that one has, in effect, constructed a statistic that is 'over-trained' to the datasets considered. The main assumption that we have made in this paper is that it reasonable to treat the standard error from each gene as a constant. This assumption appears to be reasonable for the Latin Square and technical replicate data we have examined (Figures 4 and 7). It is not, however, a perfect assumption. The distribution of *P *values observed in Figures 4 and 7 are not perfectly uniform. This assumption is clearly more reasonable, however, than the assumptions used to generate the *P *values for the standard *t *and cyber *t *tests, because *P *values produced by these tests are far from uniform (Figures 4 and 7). Genes in datasets that contain biologic replicates will, of course, exhibit a greater degree of variance than genes in the technical replicates that, by necessity, make up control datasets. Despite this, our assumptions appear to produce more reasonable results when applied to a 'real' biologic dataset than the assumptions of the cyber *t*, standard *t*, or SAM procedures (Table 1).

We have seen that even within the Latin Square dataset, cross-hybridization can affect probe sets that are annotated as null, distorting *P *values and complicating FDRs (Figure 6). Microarray experiments are prone to other artifacts, which are incompletely understood. These include saturation of probes at high signal [23], nonequilibrium hybridization conditions [24], and artifacts that arise from the dyes used in microarray experiments [25]. A recent study found that different laboratories performing the same microarray experiment on the same RNA sample obtained large differences in their results, although the results from the best performing laboratories exhibited a greater degree of correlation [26]. Given such a challenging environment, calculation of accurate FDRs remains a difficult proposition. We argue that because FDR calculations are liable to be distorted by subtle artifacts, one should err on the conservative side. We have taken a simple approach and shown that it is possible to generate a reasonable set of *P *values in a way that should become more conservative as differences increase between sets of chips. In the many cases where a conservative statistic is appropriate, we believe this approach may yield more reasonable gene lists than other currently employed methods.

## Materials and methods

### Implementation of statistics

The uniform distribution of *P *values in Figures 4, 6, and 7 was calculated as simply the inverse of the gene index. So, for example, if there were 22,000 genes in a list ordered by statistic score, then the expected *P *value for the first gene under a uniform distribution was 1/22,000. The expected *P *value for the second gene was 2/22,000 and so forth.

Background correction, quantile-quantile normalization, and RMA summary values were calculated with RMA express [27]. In cases in which data were not normalized, background subtraction was also not performed, but RMA summary values were still generated on non-normalized data with RMA express. All RMA values are reported on a log_2 _scale.

The HG-U133A Latin Square dataset was downloaded from Affymetrix (Santa Clara, CA, USA) [28]. For the Latin Square data sets, probe sets 209374_s_at, 205397_x_at, and 208010_s_at were excluded for all analyses, as instructed by the HG-U133A_tag_Latin_Square.xls spreadsheet. We also excluded any probe set not in the spike-in probe sets that started with AFFX-. This left 42 true positives and 22,182 true negatives.

For the cyber *t *algorithm we used implementations available in the R Bioconductor package with the default parameters. The cyber *t *code was downloaded from the cyber *t *web page [29]. The cyber *t *test compares arrays for genes in two conditions producing a *P *value for each gene for the null hypothesis that the mean intensity in each condition is the same. For each gene in each of the two conditions, the cyber *t *test with the default parameters calculates a weighted standard deviation as follows:

SDcyberT=10*SDWindow2+(n−1)*SD210+n−2
 MathType@MTEF@5@5@+=feaafiart1ev1aaatCvAUfeBSjuyZL2yd9gzLbvyNv2Caerbhv2BYDwAHbqedmvETj2BSbqee0evGueE0jxyaibaiKI8=vI8tuQ8FMI8Gi=hEeeu0xXdbba9frFj0=OqFfea0dXdd9vqai=hGuQ8kuc9pgc9s8qqaq=dirpe0xb9q8qiLsFr0=vr0=vr0dc8meaabaqaciGacaGaaeqabaqadeqadaaakeaaieaacaWFtbGaa8hramaaBaaaleaacaWFJbGaa8xEaiaa=jgacaWFLbGaa8NCaiaa=rfaaeqaaOGaeyypa0ZaaOaaaeaadaWcaaqaaiaa=fdacaWFWaGaaiOkaiaa=nfacaWFebWaa0baaSqaaiaa=DfacaWFPbGaa8NBaiaa=rgacaWFVbGaa83Daaqaaiaa=jdaaaGccqGHRaWkcaWFOaGaa8NBaiabgkHiTiaa=fdacaGGPaGaaiOkaiaa=nfacaWFebWaaWbaaSqabeaacaaIYaaaaaGcbaGaa8xmaiaa=bdacqGHRaWkcaWFUbGaeyOeI0Iaa8NmaaaaaSqabaaaaa@528B@

Where n is the sample size (the number of arrays in the condition), SD is the standard deviation as it is usually calculated, and SD_Window _is the average of the standard deviation of the 100 genes with the average intensity closest to the average intensity of the gene under consideration. The cyber *t *score is then calculated in the same way as the standard *t *test, with the SD_cyberT _value for each condition replacing the conventional standard deviation for each condition and an adjusted degrees of freedom of 20 + n_1 _+ n_2 _- 4 (where n_1 _is the number of array is condition 1 and n_2 _is the number of arrays in condition 2). For more details, see the cyber t report [12] and web page [29].

The Benjamini and Hochberg algorithm [18] was implemented in Java. The predicted FDR rate for a given gene in a gene list ordered by statistic *P *value is given by N × p(k)/k, where N is the number of genes in the list and p(k) is the *P *value produced by the test statistic under the null hypothesis of no differential expression for gene k in the list.

For SAM, we used the implementation in the Multiple Experiment Viewer [30,31] provided by TIGR [32].

The cdf (cumulative distribution function) function in Equation 4 was evaluated using the pnorm function in the class StatFunction.java implemented by Sundar Dorai-Raj and downloaded from the Dorai-Raj web page [33]. This function yields equivalent values to the R function pnorm with lower.tail = FALSE.

The Kolmogorov-Smirnov test was ported to Java from the Numerical Recipes in C++ text [34]. The Java port was tested against the ks.test method in R. In cases in which the Kolmogorov-Smirnov test returned a zero, the -log_10 _value was set to 200 (Figure 4) or 350 (Figures 6 and 7).

Loess regression lines were generated by the Java class Lowess. java in the package org.tigr.midas.engine distributed as part of the TIGR midas engine [32].

All statistics except cyber *t *and the results of RMA express were implemented in Java. Implementations of the equations presented in this report can be found in the supplementary materials (Additional data file 11) and at the author's web page [35].

### Etoposide treatment

Mouse embryonic stem cells were differentiated into isogenic bursting embryoid body (EB) cells or isogenic myeloid (MY) hematopoietic cells. A population of about 10^6 ^EB or MY hematopoietic cells were seeded onto five replicate dishes and expanded to obtain the appropriate number of cells per plate for treatment. About 1.5 × 10^7 ^EB or MY hematopoietic cells were exposed to either DMSO plus etoposide at a final concentration of 50 μmol/l or to DMSO (control) for 60 min. Each etoposide or DMSO control was performed on the five replicate dishes. The etoposide stock solution or DMSO (control) was diluted in Iscove's modified Dulbecco's medium supplemented with 10% non-ES-qualified fetal bovine serum. Following etoposide or DMSO (control) exposure, all samples were washed twice in 1× phosphate-buffered saline and plated in fresh medium for a recovery period of 4 or 24 hours. Following recovery all cells were washed twice in 1× phosphate-buffered saline and harvested for RNA isolation.

### RNA isolation and processing for microarrays

Control and etoposide-treated cells were pelleted by centrifugation and lysed in TRIzol Reagent (Invitrogen, Carlsbad, CA, USA; 1 ml per 10 × 10^6 ^cells) by repetitive pipetting followed by incubation at room temperature for 5 min. Total RNA was recovered by phenol-chloroform extraction and isopropyl alcohol precipitation. Extracted RNA was further purified using the RNeasy mini kit (Qiagen, Valencia, CA, USA). Biotin-labeled cDNA was prepared from the purified RNA samples using the Ovation™ Biotin RNA Amplification and Labeling System (NuGEN Technologies, Inc., San Carlos, CA, USA), in accordance with the manufacturer's protocol. Briefly, first-strand and second-strand cDNA synthesis was followed by amplification of the double-stranded DNA template. Amplified cDNA was then fragmented and labeled with biotin. Biotin-labeled cDNA was purified using the DyeEx 2.0 Spin Kit (Qiagen), and product yield and purity were determined u A260, A280, and A320 spectrophotometric measurements. Fragmented, biotin-labeled cDNA (2.2 μg) from each sample was hybridized to a GeneChip Mouse Genome 430 2.0 array (Affymetrix, Inc.). The Mouse Genome 430 2.0 array contains 45,000 probe sets used to analyze the expression level of over 39,000 transcripts from over 34,000 mouse genes. Hybridization, washing, staining, and scanning of microarrays was performed by the Gene Chip Analysis Facility in the Institute for Cancer Genetics, Columbia University Health Sciences Division, New York, USA.

## Additional data files

The following additional data are available with the online version of this manuscript.Raw data (in the form of 40 .cel files) for the Etoposide experiments described in Table 1 can be found in the supplementary materials in this paper (Additional files 1, 2, 3, 4, 5, 6, 7, 8, 9, 10 compressed with bzip2). Filenames within the zip files that start with MY indicate myeloid hematopoietic cells while filenames starting with EB indicate bursting embryoid bodies. The third character of each file indicates treatment with drug + DMSO ("E") or just control DMSO ("C"). The next character of each filename indicates the replicate number. The final characters indicate the time point. So, for example, "MYE34.CEL" indicates a myeloid hematopoietic cells ("MY") treated with drug ("E"), replicate number 3, 4 hour time point. "EBC124.CEL" indicates bursting embryoid bodies ("EB"), treated with only DMSO ("C"), replicate number 1, 24 hour time point. Additional data file 11 provides implementations of the equations presented in this report in R.

## Supplementary Material

Additional data file 1 This file contains EBC14.CEL, EBC24.CEL, EBC34.CEL, and EBC44.CEL. Click here for file

Additional data file 2 This file contains EBC54.CEL, EBE14.CEL, EBE24.CEL, and EBE34.CEL. Click here for file

Additional data file 3 This file contains EBE44.CEL, EBE54.CEL, EBC124.CEL, and EBC224.CEL. Click here for file

Additional data file 4 This file contains EBC324.CEL, EBC424.CEL, EBC524.CEL, and EBE124.CEL. Click here for file

Additional data file 5 This file contains EBE224.CEL, EBE324.CEL, EBE424.CEL, and EBE524.CEL. Click here for file

Additional data file 6 This file contains MYC14.CEL, MYC24.CEL, MYC34.CEL, and MYC44.CEL. Click here for file

Additional data file 7 This file contains MYC54.CEL, MYE14.CEL, MYE24.CEL, and MYE34.CEL. Click here for file

Additional data file 8 This file contains MYE44.CEL, MYE54.CEL, MYC124.CEL, and MYC224.CEL. Click here for file

Additional data file 9 This file contains MYC324.CEL, MYC424.CEL, MYC524.CEL, and MYE124.CEL. Click here for file

Additional data file 10 This file contains MYE224.CEL, MYE324.CEL, MYE424.CEL, and MYE524.CEL. Click here for file

Additional data file 11 Provided are implementations of the equations presented in this report in R. Click here for file

## References

[B1] Tusher VG, Tibshirani R, Chu G (2001). Significance analysis of microarrays applied to the ionizing radiation response.. Proc Natl Acad Sci USA.

[B2] Storey JD, Tibshirani R (2003). Statistical significance for genomewide studies.. Proc Natl Acad Sci USA.

[B3] Benjamini Y, Yekutieli D (2001). The control of the false discovery rate in multiple testing under dependency.. Ann Stat.

[B4] Reiner A, Yekutieli D, Benjamini Y (2003). Identifying differentially expressed genes using false discovery rate controlling procedures.. Bioinformatics.

[B5] Dabney AR, Storey JD (2006). A reanalysis of a published Affymetrix GeneChip control dataset.. Genome Biol.

[B6] Huber W, von Heydebreck A, Sultmann H, Poustka A, Vingron M (2002). Variance stabilization applied to microarray data calibration and to the quantification of differential expression.. Bioinformatics.

[B7] Li C, Wong WH (2001). Model-based analysis of oligonucleotide arrays: expression index computation and outlier detection.. Proc Natl Acad Sci USA.

[B8] Irizarry RA, Hobbs B, Collin F, Beazer-Barclay YD, Antonellis KJ, Scherf U, Speed TP (2003). Exploration, normalization, and summaries of high density oligonucleotide array probe level data.. Biostatistics.

[B9] Irizarry RA, Bolstad BM, Collin F, Cope LM, Hobbs B, Speed TP (2003). Summaries of Affymetrix GeneChip probe level data.. Nucleic Acids Res.

[B10] Irizarry RA, Wu Z, Jaffee HA (2006). Comparison of Affymetrix GeneChip expression measures.. Bioinformatics.

[B11] Allison DB, Cui X, Page GP, Sabripour M (2006). Microarray data analysis: from disarray to consolidation and consensus.. Nat Rev Genet.

[B12] Baldi P, Long AD (2001). A Bayesian framework for the analysis of microarray expression data: regularized t test and statistical inferences of gene changes.. Bioinformatics.

[B13] Choe SE, Boutros M, Michelson AM, Church GM, Halfon MS (2005). Preferred analysis methods for Affymetrix GeneChips revealed by a wholly defined control dataset.. Genome Biol.

[B14] Gaile DP.;Miecznikowski JC, Choe SE, Halfon MS (2006). Putative Null Distributions Corresponding to Tests of Differential Expression in the Golden Spike Dataset are Intensity Dependent Technical Report 06-01.

[B15] Choe SE, Boutros M, Michelson AM, Church GM, Halfon MS (2006). Correspondence: response to Dabney and Storey.. Genome Biol.

[B16] Cope L, Hartman S, Gohlmann H, Tiesman J, Irizarry RA (2005). Analysis of Affymetrix GeneChip Data Using Amplified RNA: Working Paper 84.

[B17] Qiu X, Klebanov L, Yakovlev A (2005). Correlation between gene expression levels and limitations of the empirical bayes methodology for finding differentially expressed genes.. Stat Appl Genet Mol Biol.

[B18] Benjamini Y, Hochberg Y (1995). Controlling the false discovery rate: a practical and powerful approach to multiple testing.. J Roy Stat Soc Ser B (Methodological).

[B19] Wu C, Carta R, Zhang L (2005). Sequence dependence of cross-hybridization on short oligo microarrays.. Nucleic Acids Res.

[B20] Parrish RS, Spencer HJ (2004). Effect of normalization on significance testing for oligonucleotide microarrays.. J Biopharm Stat.

[B21] Hoffmann R, Seidl T, Dugas M (2002). Profound effect of normalization on detection of differentially expressed genes in oligonucleotide microarray data analysis.. Genome Biol.

[B22] Irizarry RA, Cope L, Wu Z Feature-level exploration of the Choe *et al*. Affymetrix Genechip control dataset (Working Paper 102).. http://www.bepress.com/jhubiostat/paper102.

[B23] Naef F, Socci ND, Magnasco M (2003). A study of accuracy and precision in oligonucleotide arrays: extracting more signal at large concentrations.. Bioinformatics.

[B24] Sartor M, Schwanekamp J, Halbleib D, Mohamed I, Karyala S, Medvedovic M, Tomlinson CR (2004). Microarray results improve significantly as hybridization approaches equilibrium.. Biotechniques.

[B25] Naef F, Magnasco MO (2003). Solving the riddle of the bright mismatches: labeling and effective binding in oligonucleotide arrays.. Phys Rev E Stat Nonlin Soft Matter Phys.

[B26] Irizarry RA, Warren D, Spencer F, Kim IF, Biswal S, Frank BC, Gabrielson E, Garcia JG, Geoghegan J, Germino G (2005). Multiple-laboratory comparison of microarray platforms.. Nat Methods.

[B27] RMA Express. http://rmaexpress.bmbolstad.com/.

[B28] Affymetrix Latin Square Data.. http://www.affymetrix.com/support/technical/sample_data/datasets.affx.

[B29] Cyber-T. http://visitor.ics.uci.edu/cgi-bin/genex/cybert/CyberTReg-8.0.form.pl.

[B30] Saeed AI, Bhagabati NK, Braisted JC, Liang W, Sharov V, Howe EA, Li J, Thiagarajan M, White JA, Quackenbush J (2006). TM4 Microarray Software Suite.. Methods Enzymol.

[B31] Saeed AI, Sharov V, White J, Li J, Liang W, Bhagabati N, Braisted J, Klapa M, Currier T, Thiagarajan M (2003). TM4: a free, open-source system for microarray data management and analysis.. Biotechniques.

[B32] TM4 Microarray Software Suite. http ://www.tm4.org/mev.html.

[B33] The Virtual Home of Sundar Dorai-Raj. http://www.stat.vt.edu/~sundar/java/code/StatFunctions.html.

[B34] Press WH, Teukolsky SA, Vetterling WT, Flannery BP (2002). Numerical Recipes in C++: The Art of Scientific Computing.

[B35] Anthony Fodor's home page. http://www.afodor.net.

